# Development of molecularly imprinted polymers for the detection of human chorionic gonadotropin

**DOI:** 10.1038/s41598-025-94289-3

**Published:** 2025-03-26

**Authors:** Radvilė Zubrytė, Liliia Mavliutova, Yadiris García, Mark V. Sullivan, Nicholas W. Turner, Francesco Patitucci, Laura C. Polania, Verónica A. Jiménez, Robert Porter, Alice Mattsson, Börje Sellergren

**Affiliations:** 1 Pharmista Technologies AB, Scheelevägen 3, 223 63 Lund, Sweden; 2 Surecapture Technologies AB, Per Albin Hanssons Väg 35, 214 32 Malmö, Sweden; 3 Biofilms Research Center for Biointerfaces, Malmö University, Per Albin Hanssons Väg 35, 214 32 Malmö, Sweden; 4 Departamento de Ciencias Químicas, Facultad de Ciencias Exactas, Universidad Andres Bello, Autopista Concepción-Talcahuano 7100, Talcahuano, Chile; 5 University of Sheffield, Dainton Building, Brook Hill, Sheffield, S3 7HF Great Britain; 6 Department of Pharmacy, Health and Nutritional Sciences, University of Calabria, Arcavacata, 87036 Rende, (CS) Italy

**Keywords:** Predictive markers, Medical and clinical diagnostics, Sensors

## Abstract

Diagnostic pregnancy tests are the most widely used immunoassays for home-based use. These tests employ the well-established lateral flow assay (LFA) technique, reminiscent of affinity chromatography relying on the dual action of two orthogonal anti-hCG antibodies. Immunoassays suffer from several drawbacks, including challenges in antibody manufacturing, suboptimal accuracy, and sensitivity to adverse storing conditions. Additionally, LFAs are typically designed for single use, as the LFA technique is non-reusable. An alternative to overcome these drawbacks is to leverage molecularly imprinted polymer (MIP) technology to generate polymer-based hCG-receptors and, subsequently, non-bioreceptor-based tests. Here, we report the development of MIP nanogels for hCG detection, exploiting epitopes and magnetic templates for high-yielding dispersed phase imprinting. The resulting nanogels were designed for orthogonal targeting of two immunogenic epitopes (SV and PQ) and were thoroughly characterized with respect to physical properties, binding affinity, specificity, and sensitivity. Molecular dynamics simulations indicated a pronounced conformational overlap between the templates and the epitopes in the native protein, supporting their suitability for templating cavities for hCG recognition. Quartz crystal microbalance (QCM)-based binding tests and kinetic interaction analysis by surface plasmon resonance (SPR) revealed nanomolar dissociation constants for the MIP nanogels and their corresponding template peptides and low uptake of lutenizing hormone (LH), structurally resembling to hCG. Receptor reusability was demonstrated in the multicycle SPR sensing mode using a low pH regeneration buffer. The results suggest the feasibility of using imprinted nanogels as a class of cost-effective, stable alternatives to natural antibodies for hCG detection. We foresee applications of these binders with respect to reusable pregnancy tests and other hCG-related disease diagnostics.

## Introduction

Human chorionic gonadotropin (hCG) is a 36 kDa glycoprotein hormone produced by the placenta during early pregnancy. This protein is composed of an α and β chain forming a heterodimer, with variable glycosylation levels comprising up to ca 30% of its total molecular weight. hCG plays a pivotal role during early pregnancy by promoting the thickening of the uterine lining to support the nourishment of a growing embryo. Consequently, hCG becomes detectable shortly after fertilization^1,2^. Most pregnancy tests available on the market today demonstrate a detection accuracy of 99% when used on the day of a missed period or 14 days post-ovulation correlating with hCG levels of about 50 mIU/mL^3^. However, these levels can vary significantly among individuals. Given the frequency with which women use pregnancy tests throughout their reproductive years, they represent today one of the most commonly utilized immunoassays^4^. In other medical contexts, hCG plays a vital therapeutic role for in vitro fertilization and is a significant tumor marker across various cancers, including testicular, ovarian, and trophoblastic tumors^5^ Quantitative and specific hCG tests are thus indispensable for both pregnancy and disease management purposes. The development of corresponding tests is associated with different requirements in terms of sensitivity ranges and the need for detecting various modified forms of hCG^6^. All hCG tests are antibody-based immunoassays designed to distinguish hCG from closely related hormones such as luteinizing hormone (LH), follicle-stimulating hormone (FSH), and thyroid-stimulating hormone (TSH). While these hormones share identical α-chains with hCG, their distinct β-chains confer unique biological activities despite significant sequence homology with hCG (e.g., LH shares ~ 80% sequence identity). Accurate hCG tests depend on access to specific antibodies displaying no or minimal cross-reactivity with the other hormones^7^. Developing such antibodies, in turn, depends on a comprehensive structural mapping of all isoforms to identify proteotypic and solvent-accessible epitopes. This is further complicated by the wide range of hCG variants differing in glycosylation state and fragment size^8^. In addition, antibody-based assays suffer from inherent challenges such as complicated manufacturing, suboptimal accuracy, sensitivity to adverse storing conditions, and restriction to single use. This has led to an emerging need for robust and readily available recognition elements as sustainable alternative antibodies^9–11^.

**Fig. 1:**
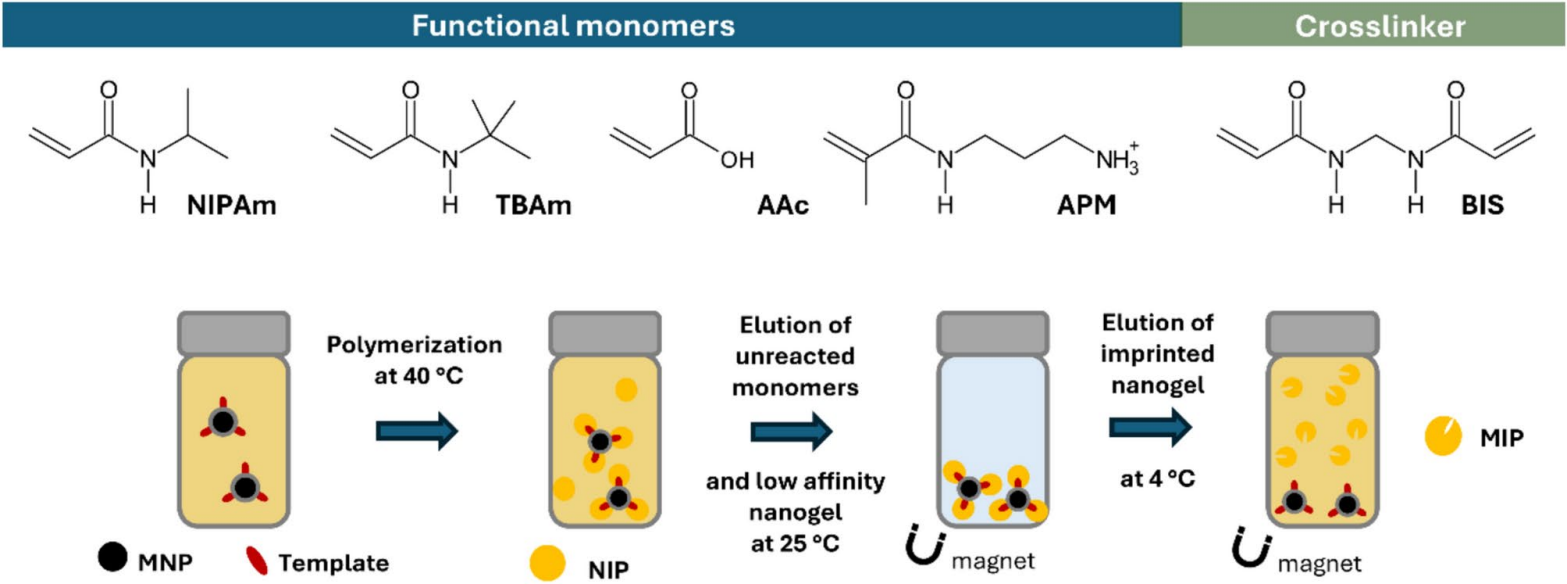
Monomers used to synthesize nanogels for hCG recognition and principle of hCG-epitope imprinting exploiting dispersions of magnetized templates to produce imprinted nanogels.

In recent years, molecularly imprinted polymers (MIPs) have emerged as promising robust synthetic antibodies for detecting fluid biomarkers^12–15^. These are prepared in the presence of a template that, post-removal, leaves behind binding sites with template-complementary shapes and functionalities. MIPs are robust and stable, negating the need for costly, temperature-controlled supply chains. Meanwhile, they exhibit molecular recognition properties comparable with antibodies, are inexpensive to produce, and are made using animal-free methods. Moreover, unaffected by harsh cleaning conditions, MIPs are reusable, potentially paving the way for more sustainable diagnostic tools^16,17^. MIPs adapted for diagnostic applications are preferably nanoparticles or nanogels produced by precipitation, emulsion, or graft polymerization,^13,14,18^ and thin-film materials^19^ that exhibit homogenous binding sites due to the spatial restrictions imposed by the limited film thickness or nanoparticle radius. Following a quasi-generic protocol, polyacrylamide-gel-based nanogels can now be manufactured to target well-established antigens^20–22^ Like antibodies, these binders can bind biological targets ranging from small to large molecules and have been implemented in sensor and assay formats. This notable progress stems from recent advances in polymer, colloid, and host–guest chemistry, particularly through the application of epitope imprinting^22–26^ combined with solid-phase^27,28^ or dispersed phase synthesis using magnetic template carriers^29–31^. In the latter methods, the template is strategically immobilized in a site-directed orientation on non-porous glass beads or magnetic nanoparticles, respectively. Subsequently, high dilution polymerization of appropriate water-soluble monomers occurs, resulting in polymers partially adhering to the surface of the template carriers. The magnetic templating method (Fig. 1) is preferred for high-yielding synthesis of epitope imprinted nanoparticles targeting hCG.

**Fig. 2:**
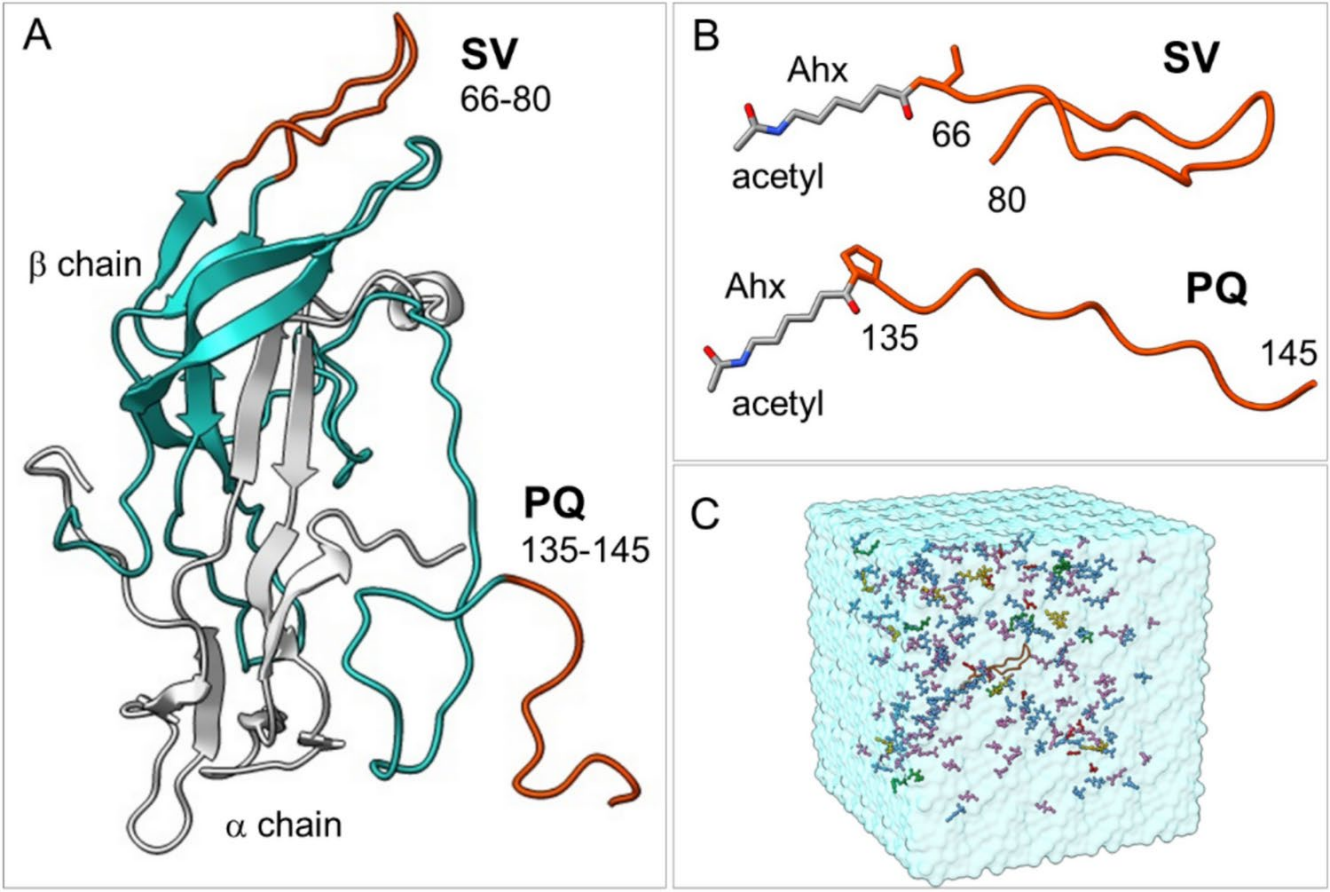
(**A**) Structure of the full hCG hormone in which the epitopes SV and PQ are highlighted. SV corresponds to the β-chain loop residues 66–80 (SIRLPGCPRGVNPVV). PQ is the C-terminal peptide of the β-chain, corresponding to residues 135–145 (PGPSDTPILPQ). (**B**) Structures of the SV and PQ peptides conjugated to the N-acetylated-6-aminohexanoyl (Ahx) moiety at the N-termini. (**C**) MD simulation box used to evaluate the conformational properties of the peptide templates (Ahx-SV or Ahx-PQ) within a pre-polymerization mixture environment. The simulated systems were built by placing the templates Ahx-SV or Ahx-PQ at the center of the box surrounded by 200 monomers in proportions consistent with the experimental setup and explicit solvent molecules.

Similar to antibody-antigen interactions, the epitope refers to a short, solvent-exposed peptide sequence (8–20 amino acids), acting as an antigenic determinant, thus constituting the specific site on the protein surface interacting with the MIP. Preferred epitopes are linear solvent-exposed C- or N-terminal sequences or internal conformationally defined loop structures. Moreover, the epitopes should be free from interfering post-translational modifications and generate adequate affinity and specificity for the target hormone, in this case, allowing detection of hCG levels in the range 10 pM – 1.0 µM and absence of cross-reactivity with luteinizing hormone (LH). Most hCG-reactive antibodies bind to assembled discontinuous epitopes and rarely to linear continuous sequences^8,32^ Two exceptions are the C-terminal peptide (βCTP) of the β-chain, aa 135–145 (PGPSDTPILPQ = PQ), and the β-chain loop structure sequence aa 66–80 (SIRLPGCPRGVNPVV = SV) (Fig. 2A). βCTP is a flexible terminal sequence lacking a defined secondary structure. Hence, its immunogenicity is limited, constraining the production of high-affinity antibodies. Given that βCTP is lacking in LH, monoclonal antibodies against this sequence show high selectivity to hCG versus LH and have found use in various commercial sandwich immunoassays^33^. Interestingly, glycosylation of this sequence at Ser138 did not seem to influence affinity for this epitope^34^. On the other hand, the β-chain loop structure sequence aa 66–80 is a highly conserved motif present in all hCG variants that has been proposed as an ideal epitope for developing a universal, single epitope hCG assay. Indeed, monoclonal antibodies recognizing this sequence show an exceptionally high target selectivity with only minor cross-reactivity with the homologous LH fragment (aa 86–100) that features an identical sequence except for Asn77, which in LH corresponds to Asp. This reflects the high performance of the corresponding monoclonal antibodies^35^.

In this report, we used these two immunogenic sequences (SV and PQ) as templates for preparing high-affinity polymer-based receptors for hCG. Molecular dynamics simulations confirmed that the templates retain their mimotopic conformations within a pre-polymerization mixture environment, thus being suitable for imprinting cavities capable of hCG recognition. The obtained MIPs recognize their templates with high affinities when used as receptors in a QCM-based or a regenerable SPR-based biosensor.

## Materials and methods

### Reagents

Ferric chloride hexahydrate (FeCl_3_·6H_2_O), sodium acetate, polyethylene glycol 6000 (PEG), sulfuric acid, glycine and sodium dodecyl sulfate (SDS) were obtained from Merck. Ethylene glycol, ammonium hydroxide 25%, triethylamine (TEA), (3-aminopropyl)trimethoxysilane (APTMS), succinic anhydride, 1-ethyl-3-(3-dimethylaminopropyl)-carbodiimide hydrochloride (EDC),* N*-hydroxysuccinimide (NHS), ammonium persulfate (APS), *N*,*N*,*N’*,*N’*-tetramethylethylenediamine (TEMED), *N*-isopropylacrylamide (NIPAm),* N*-tert-butylacrylamide (TBAm), acrylic acid (AA), tetraethyl orthosilicate (TEOS), phosphate-buffered saline (PBS), ethanolamine hydrochloride, 16-mercaptohexadecanoic acid (MHA), 11-mercapto-1-undecanol (MU), acetic acid, sodium chloride (HCl), triethylamine, 4-(2-hydroxyethyl)piperazine-1-ethane-sulfonic acid (HEPES), 2-[morpholino]ethanesulfonic acid (MES), albumin from Bovine Serum (BSA), human chorionic gonadotropin (hCG) and luteinizing hormone (LH) were obtained from Sigma-Aldrich. *N*,*N*-Dimethylformamide (DMF), acetone, hydrochloric acid (HCl) and tetrahydrofuran (THF) were purchased from VWR. *N*,*N*′-methylene-*bis*-acrylamide (MBAm) was obtained from Alfa Aesar, *N*-(3-aminopropyl)methacrylamide hydrochloride (APM) was obtained from Polysciences Inc. Ethanol was obtained from Solveco. 6-Aminohexanoyl-hCGβ_60-80_ (Ahx-SIRLPGCPRGVNPVV) and 6-Aminohexanoyl-hCGβ_135-145_ (Ahx-PGPSDTPILPQ) (> 95%) were purchased from Lifetein LLC (New Jersey, US). *N*-fluoresceinacrylamide was synthesized as reported previously^36^ Fluoresceine was purchased from TCI, and Tween 20 was obtained from AppliChem.

### Apparatus

#### Molecular dynamics simulations

MD-simulations were carried out in a computer cluster equipped with (a) 2 Supermicro Quantum TXR413-1500R servers with 2 Intel Xeon® processors E52620, and 16 NVIDIA GeForce GTX1080 GPUs and (b) 2 TensorEX TS4-1,598,415-AMB servers with 2 Intel Xeon Silver® processors and 16 NVIDIA RTC2080 GPUs.

#### Dynamic light scattering (DLS) and zeta-potential

Effective hydrodynamic diameters (*d*_*h*_) of the particles were determined by dynamic light scattering (DLS) with a Zetasizer Ultra (Malvern Panalytical) equipped with a He–Ne laser (688 nm) and set to backscatter mode, with measurements of samples performed in triplicate at 25 °C. Data was analyzed using the ZS Xplorer software.

#### Fourier-transform Infrared Spectroscopy (FTIR)

Spectra were collected using a Nicolet 6400, equipped with a DTGS detector. The *smart*iTR accessory was used to characterize dried modified magnetic nanoparticles. 500 spectra were collected at resolution 4. Compressed air was continuously run through the instrument during and before the measurements. Baseline correction and data management were performed with the OMNIC 6 software.

#### Surface plasmon resonance (SPR)

The affinity of the imprinted nanoparticles for the epitope target was investigated using a Reichert 2 SPR system (Reichert Technologies, Buffalo, USA) with an attached autosampler and degasser. The specificity of the imprinted nanomaterial was investigated by binding a non-target peptide of similar shape and size.

#### Quartz crystal microbalance (QCM) measurements

The interaction between the peptide/protein and nanogels was analyzed with Q-Sense QCM-D E4 unit equipped with a standard flow module (Biolin Scientific AB, Sweden). The sensors used for the experiments were the QCM5140TiAu120-050-Q (5 MHz) from Quartz Pro AB (Jarfalla, Sweden). All sensors were cleaned in line with the manufacturer’s recommendations.

### Experimental

#### Synthesis of magnetic nanoparticles

Magnetic nanoparticles (MNP) were obtained by solvothermal synthesis, as reported by Mahajan et al.^37^ Briefly, FeCl_3_·6H_2_O (16.6 mmol), sodium acetate (95.0 mmol), SDS (21.8 mmol) and PEG 6000 (2.7 g) were dissolved in 150 mL of ethylene glycol. The mixture was magnetically stirred at 100 °C for 30 min and then transferred to a 150 mL Teflon-lined stainless-steel autoclave. The autoclave was sealed and heated at 180 °C for 24 h. Then, the container was cooled to room temperature, and the MNPs were separated by a magnet. The solid was washed five times with deionized water, three times with 100 mL of ethanol, three times with 100 mL of acetone, and then dried in a desiccator under vacuum at 22 °C for 24 h. Subsequently, 1000 mg of MNPs were dispersed in 1 L of 80% ethanol containing 0.25 mM ammonium hydroxide, and ultrasonicated for 2 min at 50% intensity using a sonifier (BRANSON). A 0.5 M TEOS solution was then added, and the reaction mixture was shaken in an orbital shaker for 6 h. The obtained nanoparticles were washed with deionized water until neutral pH, followed by three washes with 100 mL of ethanol, and dried in a desiccator over activated silica at 22 °C for 24 h. The produced silica-coated nanoparticles (MNP@Si,1000 mg) were surface-functionalized by sonication in a bath sonicator for 2 h with APTMS (57 mM) in 75% ethanol. The resulting primary amine-modified MNPs (MNP-NH2) were washed three times with ethanol and dried in a 37 °C oven overnight. Particles were further functionalized with succinic anhydride (2.1 M) in DMF by sonication in a bath for 3 h. The final product (MNPs-COOH) was washed ten times with deionized water, three times with ethanol, and dried at 37 °C oven.

#### Template conjugation to magnetic nanoparticles (MNPs)

MNPs-COOH (100 mg) was placed into a 20 mL vial along with 6 mL of deionized water at room temperature, followed by sonication for 30 s twice at 35% amplitude. The carboxylic groups on the surface of the magnetic nanoparticles were activated by adding 2 mL of freshly prepared EDC solution (100 mg·mL^-1^) and 2 mL of NHS solution (62.5 mg·mL^-1^) in deionized water. The mixture was then shaken on an orbital shaker for 1 h at room temperature. Afterward, the activated magnetic nanoparticles were washed twice with 10 mL of deionized water and once with 5 mL of 10 mM PBS (pH 7.4). The immobilization of Ahx-PQ and Ahx-SV was carried out by adding 5 mL of epitope solution (0.50 mg·mL^-1^ in 10 mM PBS, pH 7.4) to the vial, which was then shaken at room temperature overnight. Subsequently, the peptide conjugation was assessed via the fluorescamine test.^38,39^ A portion of the initial supernatant (100 μL) from the reaction was mixed with 25 μL of Fluorescamine solution (0.50 mg·mL^-1^ in acetone). Fluorescence was then measured (λ_ex_ = 400, λ_em_ = 510 nm), and the specific amount of conjugated peptide was calculated from the reduced signal upon solution depletion. To remove any excess epitope, the MNPs were collected using an external magnet, rinsed with PBS and deionized water, then suspended in 5 mL of deionized water and stored at 4 °C.

#### Preparation of molecularly imprinted nanogels using magnetic templates

Monomer feed ratios used to synthesize the epitope-imprinted MIPs are reported in Table S2. Synthesis of MIP-PQ was conducted as follows. A pre-polymerization mixture with a total monomer concentration of 10 mM was prepared by dissolving NIPAm (5.43 mg, 48 μmol), BIS (0.31 mg, 2 μmol), TBAm (5.09 mg, 40 μmol dissolved in 1 mL of ethanol), APMA (1.79 mg, 10 μmol), and 0.4 mg of *N*-fluoresceinylacrylamide (dissolved in ethanol) in deionized water (9 mL) in a 20 mL vial. The pre-polymerization mixture was homogenized for 30 min, followed by the addition of 1 mL of 25 mg·mL^-1^ MNP-SV or MNP-PQ dispersion. The mixture was stirred at room temperature under N_2_ atmosphere for 15 min. Then, 0.26 mmol of ammonium persulfate (APS) and 0.40 mmol of *N,N,N’,N’*-tetramethylethylenediamine (TEMED) were added and the dispersion was left overnight at 40 °C under constant stirring (480 rpm). Afterward, the particles were collected using an external magnet and washed with deionized water (5 × 10 mL) at 40 °C to remove unreacted functional monomers and low-affinity nanoparticles. Finally, the high-affinity imprinted nanogels were obtained by adding 4 mL of 1 mM PBS to the magnetic particles and leaving at 4 °C for 4 h. The elution step was performed by collecting the first 4 mL, and this process was repeated once on the magnetic nanoparticles by adding an additional 1 mL of PBS and incubating overnight at 4 °C. Thus, the elution process resulted in a total volume of 5 mL of imprinted nanogel solution. Concentrations of the nanogel solutions were calculated by taking 400 µL of the solution (in triplicate) and evaporating to dryness. The mass of the dried particles was then measured, and the amount was multiplied by 2.5 to give the concentration in μg mL^−1^.

#### DLS measurements

To evaluate the hydrodynamic size of nanogels, a 100 μg mL^−1^ dispersion of the MIP nanogels in water was prepared. The sample was sonicated in a bath sonicator for 30 min and left at 4 °C for 2 h. To evaluate MNP synthesis and surface modifications, a 50 µg mL^-1^ suspension of the MNPs was prepared in water. Before the measurement samples were sonicated 3 times for 10 s at 50% amplitude. Size and zeta potential measurements were done in triplicates.

### SPR experiments

#### Immobilization of MIP nanogels onto the SPR sensor surface

A carboxymethyl dextran hydrogel-coated Au chip (Reichert, USA) was preconditioned within the SPR using a running buffer consisting of PBS (0.010 M) and 0.010% Tween 20 at pH 7.4, at a flow rate of 10 µL min^-1^. The carboxylic acid groups on the dextran chip were activated with an injection of 1 mL of aqueous solution containing 40 mg EDC and 10 mg NHS passed over the chip (6 min at 10 µL min^-1^). The MIP nanogels (approximately 300 µg), were activated by dissolving in 1 mL of 10 mM sodium acetate in PBST solution. This was injected over the left channel (working channel) of the chip for 1 min. The amine groups of the MIP nanogels react with the functionalized surface of the chip, leading to the covalent immobilization of the nanoparticles. A quenching solution of ethanolamine (1 M at pH 8.5) was injected over both channels (working and reference) for 8 min, followed by a continuous flow of PBST at 10 µL min^-1^. All injections were taken from a stable baseline.

#### Kinetic interaction analysis

The kinetic analysis for the affinity of the target peptide to the MIP nanogels was performed in a set pattern of a 2 min association (PBST with the analyte in a concentration range of 4–64 nM), 5-min dissociation (PBST only) and a regeneration cycle (regeneration buffer 10 mM Glycine–HCl, pH 2 for 1 min) followed by a final stabilization cycle (PBST for 1 min). An initial injection of blank PBST was used as the first run, with increasing analyte concentration for subsequent runs. After the analyses were completed, signals from the reference channel were subtracted from signals from the working channel. Selectivity of the MIP nanogels was investigated by repeating the kinetic analysis, but with a non-target analyte with the same concentration range (4–64 nM). All experiments were performed in triplicate (n = 3). The SPR responses were fitted to a 1:1 Langmuir fit bio-interaction (BI) model using the Reichert TraceDrawer software. The association rate constants (k_a_), dissociation rate constants (k_d_), and maximum binding (B_max_) were fitted globally, whereas the BI signal was fitted locally. Equilibrium dissociation constants (K_D_) were calculated by k_d_/k_a_. For each MIP nanogel/analyte epitope combination, a calibration curve was generated across the concentration range 4–64 nM, using the SPR fitted curve maxima. From this information, a theoretical limit of detection (LOD) was calculated.

### QCM experiments

#### Sensor chip modification

After washing with piranha solution (H_2_SO_4_:H_2_O_2_, 4:1), a gold-coated QCM sensor chip was dried with N_2_. The chip, cleaned via plasma cleaner for 5 min, was then placed in a petri dish and immersed in 4 mL of a thiol solution composed of 1 mM 16-Mercaptohexadecanoic acid (MHA) and 1 mM 11-Mercapto-1-undecanol (MU) in absolute ethanol:acetic acid (ratio 9:1) for 18 h at room temperature in the dark. Afterward, the chip was washed three times with absolute ethanol and 10 mM MES buffer (pH 6) and dried with N_2_. To activate the carboxylic group of MHA, 100 μL of a sulfo-NHS:EDC mixture (1:1, 100 mg·mL^-1^ in MES buffer) was introduced for 30 min, followed by washing with 10 mM PBS and drying with N_2_. The immobilization of the epitope was conducted using a standard solution of Ahx-PQ or Ahx-SV prepared at a concentration of 1 mg·mL^-1^ in 10 mM PBS (pH 7,4). Immobilization of hCG was carried out using a protein solution at 10 μg·mL^-1^ in PBS. The protein was injected into a QCM-D instrument to verify the bound amount. Additionally, to confirm the specificity of imprinted nanogels, the same procedure was conducted for LH.

#### QCM measurements

The experiments aimed at examining the binding of imprinted nanogels with PQ, SV and hCG were carried out utilizing various concentrations of MIP-PQ or MIP-SV (12.5, 25, 50, 100 μg·mL^-1^) prepared through the same procedure employed for DLS measurements. The fundamental frequency was approximately 5 MHz and the baseline frequency signal was calibrated by injecting a running buffer solution composed of 1 mM PBS and Tween 20 (0.005%, v/v) for 1 h at a flow rate of 10 μL·min^-1^ until the frequency signal response stabilized within ± 0.2 Hz/5 min. The nanogel solutions were introduced using a flow rate of 100 µL/min for 3min followed by 10 µL/min for 20 min. The frequency variations attributed to binding with analytes were recorded in accordance with the frequency signal response. To evaluate the specificity of MIP-PQ and MIP-SV in detecting hCG and LH, the proteins were immobilized on QCM sensor chips, and the coupling was analyzed using the same dilutions as before. The apparent molarities of nanogels were determined using Eq. 1.

1 $$\left[M\left(app\right)\right] = \frac{6}{\pi {N}_{A}{d}^{3}\rho } X$$

where, *d* is the hydrodynamic diameter of particles, N_A_ is Avogadro’s constant (6.023·10^23^ mol^-1^), X is the nanogel concentration in g·mL^-1^ (1·10^–4^ g·mL^-1^) and ρ is the polymer density. The results revealed an apparent molarity of ~ 40 nM.

#### Molecular dynamics simulations

The coordinates of the full hCG protein were retrieved from the Protein Data Bank server (PDB code 1HRP)^40^ The missing protein segments were built using the Chimera X interface to Modeller tools. The coordinates of the epitopes SV and PQ were obtained from residues 60–80 and 135–145 of the β-chain, respectively. Both epitopes were modified at the N-terminal end by attaching an N-acetylated-6-aminohexanoyl (Ahx) moiety. The modified epitopes were placed at the center of a cubic box of 80 Å side and surrounded by 200 randomly located monomers in proportions consistent with the formulations reported in this work using the PACKMOL software^41^ The simulated mixtures were neutralized with Na^+^ or Cl^-^ ions and solvated in a cubic box of explicit OPC waters considering an outer layer of 5 Å measured from the outermost atoms. The ff19SB force field was used to model the peptidic segments, whereas the Ahx moieties were modeled using parameters consistent with the gaff2 force field with AM1-BCC charges. MD simulations were carried out using the following protocol: (a) 1500 steepest descent minimization steps followed by 3500 conjugate gradient minimization steps for water molecules relaxation, (b) 1500 steepest descent minimization steps followed by 6500 conjugate gradient minimization steps for the entire system, (c) 500 ps of progressive heating from 0 to 300 K in a canonical ensemble (NVT) with a constant number of particles (N), volume (V), and temperature (T), (d) 10 ns of equilibrium at 300 K to ensure density equilibration in a isothermal–isobaric ensemble (NPT) with a constant number of particles (N), pressure (P), and temperature (T), (e) 20 ns of NPT equilibrium at 300 K, and finally (f) 150 ns of NPT production dynamics at 300 K and 1 bar from which production data were collected. During MD simulations the cutoff for non-bonded terms was 10 Å, long-range electrostatics were treated using the Particle-Mesh Ewald approach, and the SHAKE algorithm was employed to constrain all bonds involving hydrogen. Positional restraints were applied to the terminal acetyl group of the Ahx moiety throughout the simulation protocol to mimic the immobilization of the epitopes to the solid support through Ahx. All Calculations were carried out using the pmemd.CUDA software implemented in AMBER20. Trajectory analysis was carried out with the CPPTRAJ^42^ and VMD^43^ software.

## Results and discussion

This study reports the development of molecularly imprinted polymer nanogels (MIPs) exploiting an epitope-based approach for hCG detection. To this aim, we focused on the 15aa sequence corresponding to the β3-loop of the β-subunit from residues 60 to 80 (SIRLPGCPRGVNPVV = SV) and the C-terminal undecapeptide of the β-chain from residues 135 to 145 (PGPSDTPILPQ = PQ) as templates. To confirm that these sequences properly mimic the three-dimensional conformation of the corresponding protein epitopes, we performed molecular dynamics (MD) simulations.

### Molecular dynamics simulations

MD simulations were used to investigate the conformational properties of the SV and PQ segments in the full hCG protein and in the context of pre-polymerization formulations. This is relevant to confirm whether the selected templates preserve the structural features required for imprinting molecular cavities capable of efficiently recognizing the full protein. To that end, we conducted MD simulations on the structures of the SV and PQ peptides conjugated with the Ahx moiety (Ahx-SV and Ahx-PQ, respectively) (Fig. 2B) and surrounded by a mixture of functional monomers and explicit solvent molecules in proportions consistent with the experimental setting reported in this work (Fig. 2C). Parallel simulations were carried out on the structure of the full hCG protein in water to evaluate the intrinsic dynamic properties of the SV and PQ epitopes.

**Fig. 3:**
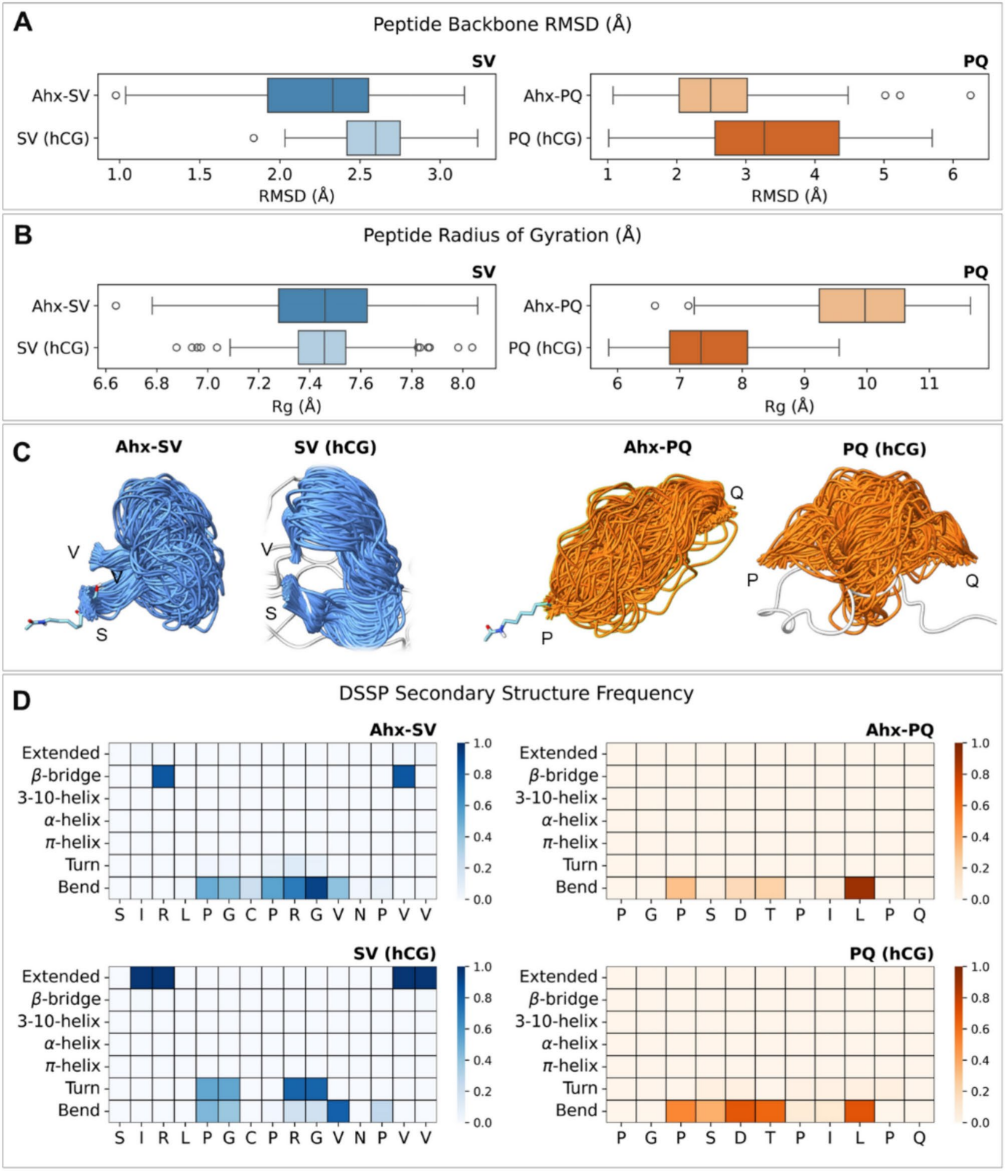
(**A**) Root-mean-square deviation RMSD (Å) distributions calculated for the peptide backbone in Ahx-SV and Ahx-PQ and the corresponding epitopes in the hCG protein. Data was collected from the analysis of 150 ns MD simulations in pre-polymerization mixtures (Ahx-SV and Ahx-PQ) or water (full hCG), using the coordinates of the SV and PQ epitopes in the 1HRP crystallographic structure as reference models. (**B**) Peptide radius of gyration R_g_ (Å) for the SV and PQ sequences in Ahx-SV, Ahx-PQ, and the full hCG protein calculated from 150 ns MD trajectories. (**C**) Structural representations of the backbone conformations achieved by the SV and PQ sequences in Ahx-SV, Ahx-PQ, and the full hCG protein throughout 150 MD trajectories. Structures are displayed from MD trajectories aligned to the first and last amino acids of each sequence. (**D**) Heatmaps for the frequency of secondary structure assignations according to DSSP analysis on SV and PQ sequences in Ahx-SV, Ahx-PQ, and the full hCG protein throughout 150 MD trajectories.

The global conformational properties of the template peptides Ahx-SV and Ahx-PQ and the corresponding epitopes in the hCG protein were evaluated from root-mean-square deviation (RMSD) calculations on the peptide backbone atoms throughout each simulation run, using the epitope coordinates in the 1HRP crystallographic model as the reference structure (Fig. 3A). RMSD data indicate that the template peptides Ahx-SV and Ahx-PQ have similar mobilities as the corresponding epitopes in the full protein, with PQ being more flexible than SV. The size and compactness of each peptide were evaluated from radius of gyration (R_g_) calculations along each MD trajectory (Fig. 3B). SV has very similar R_g_ distributions in the Ahx-SV template and in the full hCG protein, whereas PQ has slightly larger R_g_ values in the Ahx-PQ system, suggesting more extended conformations than in the hCG epitope. The structural consistency between the SV peptide in Ahx-SV and the full protein was confirmed by the visual inspection of the conformations achieved by the sequence in their MD trajectories (Fig. 3B,C). SV retained the loop structure required to imprint cavities suitable for hCG recognition, which is a relevant outcome to support the choice of this segment as a template for the synthesis of molecularly imprinted nanogels. In the case of PQ, the peptide adopts more extended conformations in the Ahx-PQ than in the hCG protein, as inferred from R_g_ data. Regarding the intrinsic arrangement of the peptide sequences, DSSP secondary structure analysis confirmed that SV and PQ are highly disordered peptides that exist mostly in turn and bend states both in the Ahx-conjugated templates and the full hCG protein (Fig. 3D). This conformational plasticity is relevant for the epitopes to adapt to imprinted cavities of variable size and shape, which supports the choice of SV and PQ as template peptides in our experimental design.

**Fig. 4:**
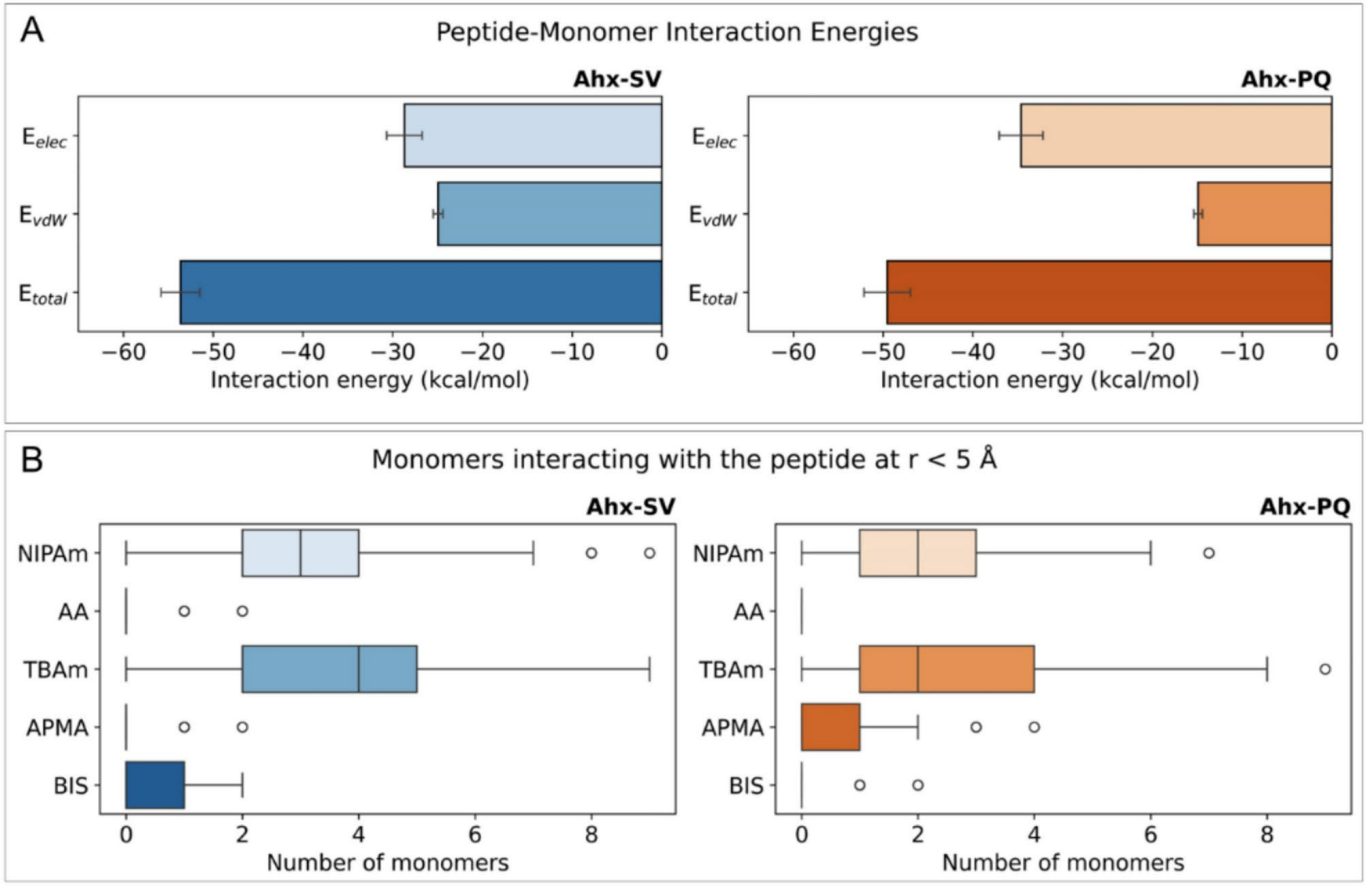
(**A**) Peptide-monomer interaction energies calculated under the LIE approach for the Axh-SV and Ahx-PQ peptides with their surrounding monomer mixtures. The total interaction energy (E_total_) corresponds to the sum of electrostatic (E_lec_) and van der Waals (E_vdW_) energy contributions. Energy terms are reported as mean values (kcal/mol) with their corresponding standard errors. (**B**) Distribution data for the number of monomers that interact with the Axh-SV and Ahx-PQ peptides at distances ≤ 5 Å throughout the MD trajectories.

Besides conformational features, we used MD simulations to estimate the strength of peptide-monomer interactions within the pre-polymerization mixtures under a LIE approach. This method decomposes the total interaction energy between a molecule and its surrounding environment (E_total_) as the sum of electrostatic (E_lec_) and van der Waals (E_vdW_) energy terms. Energy components were calculated between all atoms in the peptides with all atoms in the surrounding monomer mixtures with a distance cutoff of 12 Å along the entire MD trajectories (Fig. 4A). For Ahx-SV, the interaction with the monomer mixture is almost equally driven by the contributions from E_lec_ and E_vdW_ terms. On the other hand, the interaction of the monomer mixture with Ahx-PQ shows a stronger E_lec_ component over E_vdW_. A possible explanation for this effect arises from the presence of the highly polar SDT triad in PQ, which can engage in strong electrostatic or hydrogen bonding interactions with opposite-charged moieties of the surrounding monomers. Added to the energetics of peptide-monomer interactions, we examined the organization of the pre-polymerization mixtures by counting the number of monomers interacting with the peptides at distances ≤ 5 Å throughout the MD trajectories (Fig. 4B). Distribution data reveals that the monomer organization mostly relies on TBAm and NIPAm, which are the monomers with higher proportions in both monomer mixtures.

### Template magnetization

Magnetic nanoparticles were synthesized via solvothermal method and subsequently surface-functionalized to yield carboxyl-terminated magnetic nanoparticles (MNP-COOH). DLS analysis (Table 1) and FTIR (Fig. S3) confirmed the successful surface modifications. The MNP-COOH particles were conjugated with the peptides SV and PQ via their Ahx linkers, resulting in the template-conjugated magnetic nanoparticles referred to as MNPs-SV and MNPs-PQ. The surface coverages of the peptides were estimated to be 8.3 and 11.7 µg·g-1 for Ahx-SV and Ahx-PQ, respectively, which are comparable to coverages reported elsewhere.^22,30,31^ DLS measurements (Table 1) confirmed that the template-conjugated MNPs maintained nanoscale hydrodynamic radii (ca 280 nm) and exhibited Zeta potential changes agreeing with the net charges of the epitopes (Table S1).

**Table 1:** Hydrodynamic radii (nm), PI, and Z-potential (mV) of the MNPs and MIPs reported in this work.

System	Average size (d.nm)	Polydispersity index	Z-potential (mV)
MNP	189 ± 1	0.080 ± 0.01	17.7 ± 0.9
MNP@Si	284 ± 8	0.17 ± 0.02	-27.7 ± 0.1
MNP-NH_2_	274 ± 1	0.16 ± 0.02	25.9 ± 0.6
MNP-COOH	259 ± 4	0.090 ± 0.04	-33.1 ± 1.2
MNP-PQ	432 ± 8	0.22 ± 0.02	-40.6 ± 0.4
MNP-SV	670 ± 23	0.22 ± 0.02	-25.7 ± 0.2
MIP-SV	41 ± 14	0.22 ± 0.02	-22.0 ± 9
MIP-PQ	100 ± 2	0.16 ± 0.03	15.0 ± 4

### MIP synthesis

MIPs were synthesized by free radical polymerization of acrylamide monomers in the presence of the template-conjugated MNPs. The monomers were rationally selected based on previous designs, to enable diverse intermolecular interactions with the immobilized peptides. The template peptides SV (SIRLPGCPRGVNPVV) and PQ (PGPSDTPILPQ) comprise 15 and 11 amino acids, respectively. SV contains two positively charged and eight hydrophobic amino acids reflected in both a high pI and Gravy index (10.9 and 0.30). On the other hand, PQ features one negatively charged and seven hydrophobic residues resulting in a low pI and Gravy index (3.1 and -0.64) (Table S1). Building upon previously reported examples, poly-NIPAm-based nanogels were synthesized using a template matching ratio of the charged monomers *N*-(3-aminopropyl)methacrylamide hydrochloride (APM) and acrylic acid (AA) in addition to the hydrophobic functional monomer *N*-tert-butylacrylamide (TBAm) (Table S2). Additionally, *N,N’*-methylenebisacrylamide (BIS) (2%) was used as a cross-linker, and the fluorescent functional monomer *N*-fluoresceinylacrylamide (FITC-AAm) was doped to allow fluorescent tracking of the nanogels^30,44^ The polymeric nanogels were purified by magnetic decantation with pure water, resulting in a mass yields of 633 mg SV-MIP and 153 mg PQ-MIP per gram of MNP carrier. This confirms that mass yields can be dramatically enhanced (> > 100x) using high surface area MNPs as template carriers^30,31^ The average size, polydispersity index, and Z-potential of the MIP nanogels assessed through DLS (Fig. S1, Table 1) confirmed their nanoscale dimensions although with a minor aggregation tendency, notably in the case of MIP-SV (Fig. S1). The latter display a second, albeit much smaller, distribution peak, which we attribute to slight aggregation of the nanogels^31^ Nevertheless, the DLS size distribution supports that this synthetic method for the production of imprinted nanogels generally produces regular homogenous nanoparticles. Any potential aggregation issues was taken into account, and to prevent any potential issues during immobilisation onto the SPR chip surface, the dried nanogels were redissolved in the running buffer of PBST (PBS containing 0.01% Tween20). The MIPs were also characterized by FTIR spectroscopy as shown in Fig. S2. FTIR analysis revealed the characteristic amide bands for both MIP-SV and MIP-PQ at 1640 cm⁻^1^ (amide 1), 1536 cm⁻^1^ (amide 2), and 1223 cm⁻^1^ (amide 3). Additionally, the symmetric and asymmetric CH stretch bands are present at 2933 cm⁻^1^ and 2875 cm⁻^1^, respectively.

### Kinetic interaction analysis by surface plasmon resonance (SPR)

For a preliminary assessment of the MIP recognition properties, we performed a kinetic interaction analysis using surface plasmon resonance (SPR). Deposition of the nanogels onto the surface of the SPR chip was achieved through amide coupling on pre-functionalized gold surfaces with a carboxymethyl dextran layer^17,31^. The carboxyl groups on the chip surface were activated by EDC/NHS and conjugated with excess nanogels through their amine functionalities provided by the side chains of the APM moieties. Ethanolamine was then used to deactivate any unreacted carboxyl groups left on the SPR chip surface after nanogel immobilization while washing away the fraction of unbound nanogel. This deposition method is expected to leave a single nanogel layer on the chip surface with maximum coverage of available binding sites. The nanogel-modified SPR chips were then tested in the multicycle mode with solutions of increasing concentrations of the target peptides SV and PQ with intermediate regeneration of the sensors using a pH 2 glycine buffer. The SPR plots showing the RU changes occurring upon injections of five different concentrations of the target peptides are displayed in Fig. S3. The overall equilibrium dissociation constant (*K*_*D*_) values for the target interacting with their nanogels were calculated from the curves using a 1:1 kinetic model, leading to K_D_:s of 85 nM and 92 nM for PQ and SV, respectively (Table 2). These results are consistent with previously published values for nanogels imprinted for peptides and demonstrate the capacity of MIP-SV and MIP-PQ to rebind their template epitopes with high affinity^31^. Cross-reactivity SPR experiments with swapped templates (SV for MIP-PQ-conjugated sensors and PQ for MIP-SV-conjugated sensors) resulted in nearly fourfold increases in *K*_*D*_ values, which support the selectivity of the nanogels for their corresponding target peptides. Nevertheless, both SV and PQ exhibit non-specific nanomolar dissociation constants to the conjugated sensors, which might arise from intermolecular interactions involving the nanogel surface or the highly polar groups available from the ethanolamine or uncovered carboxymethyl dextran layers on the SPR chip.

**Table 2:** Calculated equilibrium dissociation constant (K_D_) of the nanogels obtained from SPR data presented in Fig. S3. All experiments were performed under ambient conditions and with three replicates.

MIP	K_D_ (nM)	
	PQ	SV
MIP-PQ	85.0 (± 1.0)	465 (± 25)
MIP-SV	346 (± 42)	92.0 (± 2.0)

### Nanogels binding affinity towards target peptides and proteins assessed by quartz crystal microbalance (QCM)

QCM was used for quantifying the interaction between the nanogels and their epitope templates, the full hCG protein, and the decoy protein LH^20,21,45^ For the measurements, we adopted the most commonly reported technique based on template-modified sensor chips and the MIP injected in the running buffer. First, the sensor surface was modified with a mixed Self-Assembled Monolayer (SAM) of mercaptohexadecanoic acid (MHA) and mercaptoundecanol (MU), the latter serving as a filler for improved ligand accessibility (Fig. S4). Subsequently, the ligands (SV, PQ, hCG or LH) were immobilized via EDC/NHS catalyzed coupling through their amine functionalities. The progression of this step was monitored through changes in resonant frequency (ΔF), with estimated surface coverages of 4.9·10^–11^ mol·cm^-2^ and 4.1·10^–11^ mol·cm^-2^ for PQ and SV, respectively. For hCG and LH, the estimated immobilization ratios were 1.2·10^–12^ mol·cm^-2^ and 1.3·10^–12^ mol·cm^-2^, respectively (Fig. S5).

**Fig. 5:**
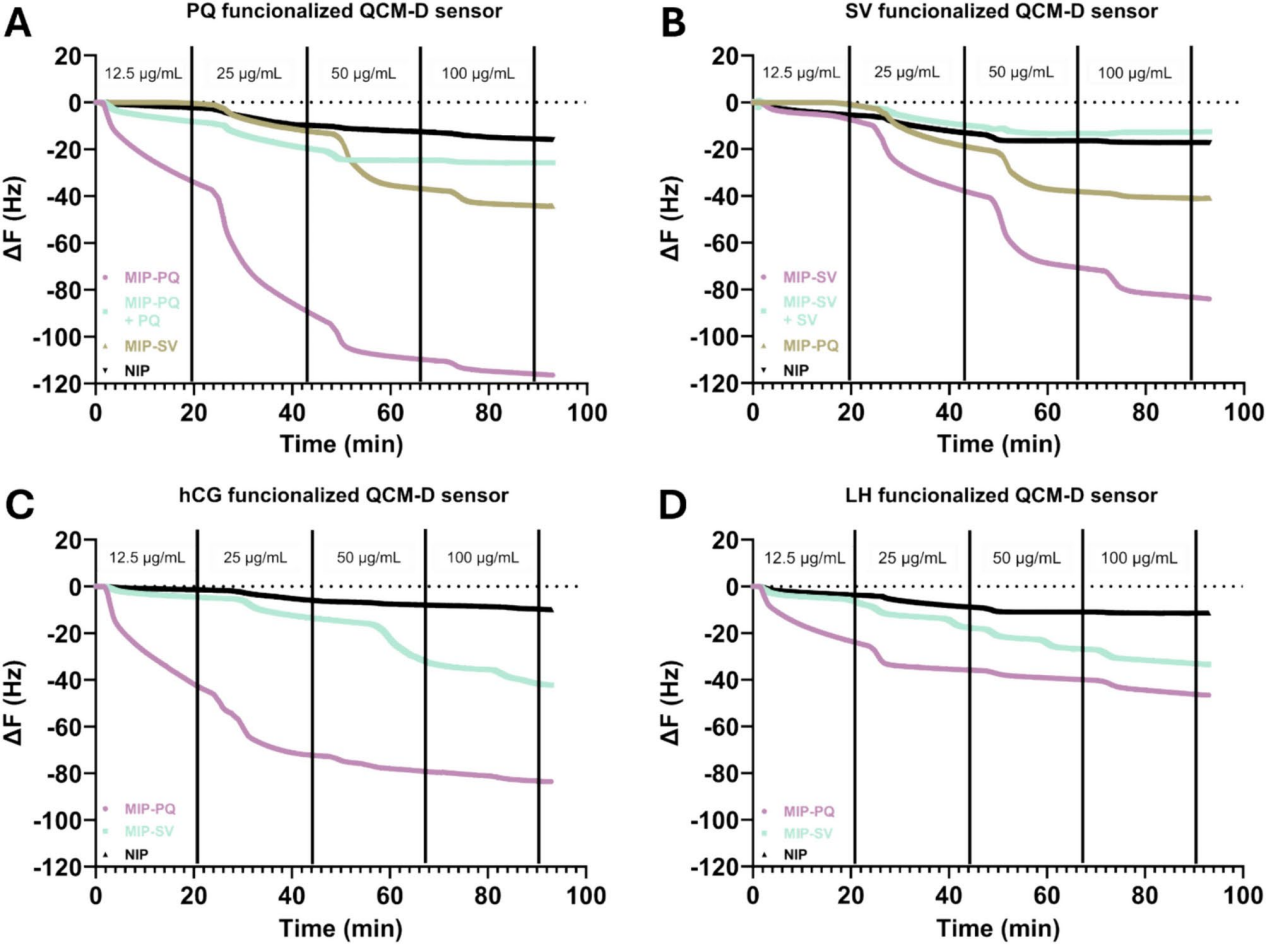
Real-time resonant frequency changes ΔF (Hz) after repeated injections of MIP-PQ, MIP-SV, and NIP dispersions of increasing concentration on functionalized QCM-D sensor chips modified with (**A**) PQ, (**B**) SV, (**C**) hCG, and (**D**) LH. Figures (**A**) and (**B**) also show the frequency changes upon injecting nanogels dispersions pre-incubated with 0.1 mg·mL^-1^ peptide solutions (MIP-PQ + PQ and MIP-SV + SV, respectively).

**Fig. 6:**
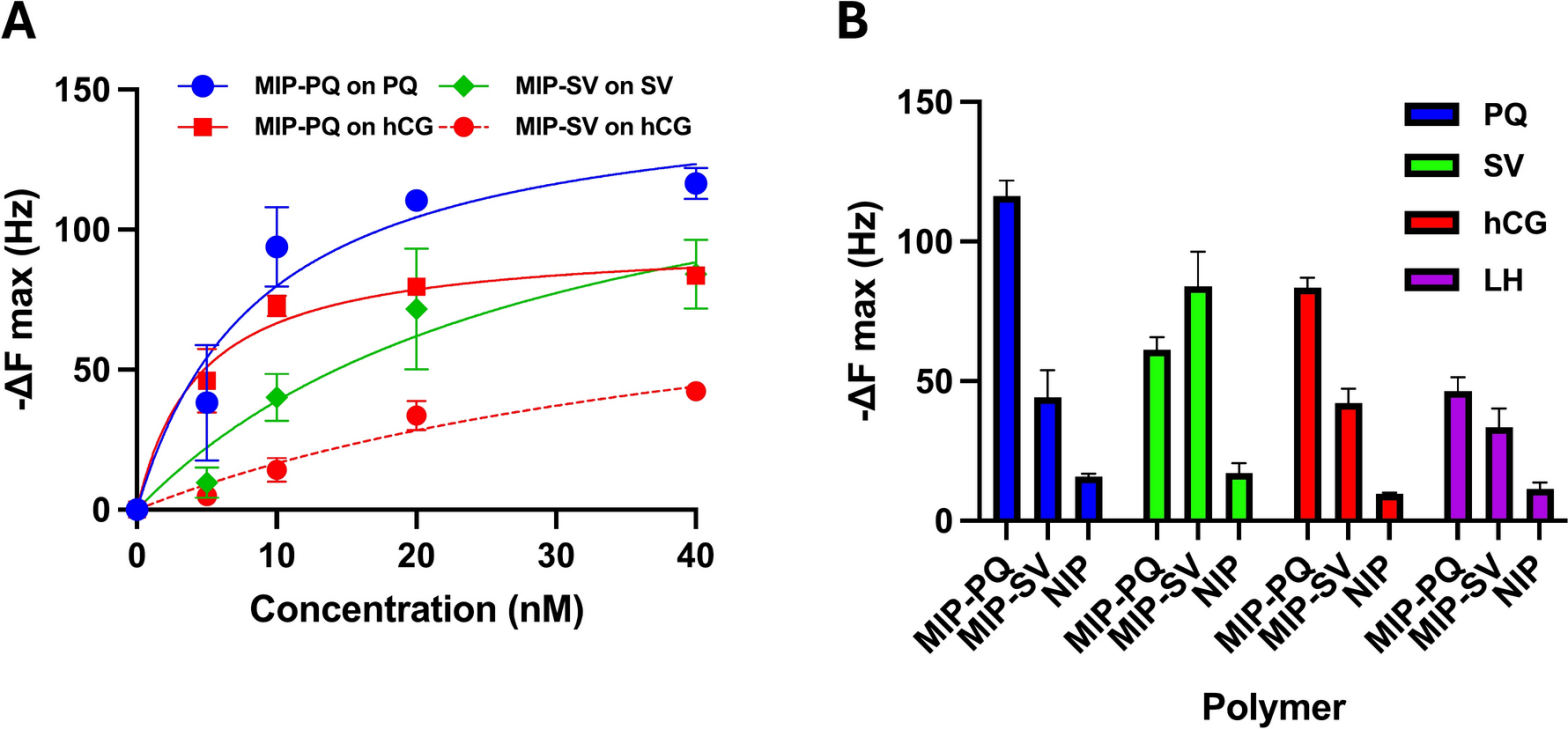
(**A**) Apparent non-equilibrium binding isotherms of MIP-PQ on a PQ (blue circles) and hCG (red squares) functionalized sensor chip and MIP-SV on an SV (green diamonds) and hCG (red circles) functionalized sensor chip, obtained from the QCM experiments in Fig. 5. The data were fitted to a 1:1 Langmuir adsorption model to determine the apparent dissociation constants given in Table 3. (**B**) Maximum frequency changes registered for the different sensor chips at a nanogel concentration of 40 nM.

The epitope-functionalized QCM chips were first used to assess the binding of MIP-PQ and MIP-SV to their corresponding templates (Fig. 5A,B). Increasing concentrations of colloidal MIP solutions were introduced at predetermined time intervals, and the frequency change (ΔF), which is proportional to mass uptake on the sensor surface, was monitored in real time. Introducing the MIPs elicited pronounced frequency decreases relative to the injection of dispersions of non-imprinted nanoparticles (NIP) of identical chemical composition. This highlights the relevance of the SV- and PQ-imprinted cavities to trigger the template recognition over non-specific intermolecular interactions on the nanogel surface. Parallel experiments with swapped nanogels-chips pairs (MIP-SV against PQ-functionalized chips and MIP-PQ against SV-functionalized chips) also resulted in significant frequency changes compared to NIPs, which suggests the presence of non-specific binding cavities capable of hosting flexible peptide moieties. Apparent dissociation constants (*K*_D_) and maximum frequency shifts (∆Fmax) were estimated for each system by fitting non-equilibrium frequency changes against injected concentrations to a Langmuir adsorption isotherm model (Fig. 6). This analysis yielded a *K*_D_ of ca 8.9 and 30 nM respectively for MIP-PQ and MIP-SV for their corresponding peptide templates (Table 3). These values are in the same order of magnitude as those determined by SPR, with expected variations arising from the QCM-associated errors due to a lack of equilibrium during data acquisition, making SPR more robust for kinetic and affinity characterization. To demonstrate the binding specificity, we performed a competitive inhibition test by pre-incubating the nanogels with soluble PQ or SV peptides (Fig. 5A,B, labels MIP-PQ + PQ and MIP-SV + SV). Pre-incubation significantly reduced the subsequent binding of MIP-SV and MIP-PQ to the template-functionalized sensors, implying that epitope occupancy of imprinted sites inhibits further nanogel interactions. These results provide compelling evidence that the MIPs achieve the design goal of selective, epitope-specific binding to the templates.

**Table 3:** Calculated equilibrium dissociation constant (K_D_) of the nanogels interacting with their corresponding epitopes and hCG from the QCM data presented in Fig. 6A. All experiments were performed under ambient conditions and with three replicates.

**MIP**	**Epitope**			**hCG**		
	K_D_ (nM)	ΔFmax	R^2^	K_D_ (nM)	ΔFmax	R^2^
MIP-PQ	8.9 ± 4.3	151 ± 25	0.95	4.4 ± 1.2	96 ± 6.0	0.99
MIP-SV	30 ± 19	154 ± 53	0.95	49 ± 35	98 ± 45	0.96

Additional QCM experiments were conducted to assess the nanogel affinity for hCG and LH proteins. To that aim, the two proteins were immobilized on the sensor chips. Following concentration-dependent exposures of MIP-SV and MIP-PQ, the hCG-functionalized sensor led to pronounced frequency drops, significantly exceeding those of NIPs (Fig. 5C). Apparent *K*_D_ values for hCG were estimated to 4.4 nM for MIP-PQ and 49 nM for MIP-SV (Table 3) (Fig. 6A). Parallel experiments using the LH-modified sensor chip produced significantly lower resonance frequency drops (Fig. 5D,6B), highlighting the selectivity of the MIPs towards the target protein hCG. The combined outcomes of QCM experiments with hCG- and LH-modified sensors suggest a higher protein discrimination capacity for MIP-PQ, which can be attributed to either an unfavorable protein immobilization masking the SV epitope or an intrinsically lower affinity of MIP-SV for the epitope.

## Conclusions

A solid-phase epitope-based synthetic approach was applied to obtain water-soluble molecularly imprinted nanoparticles for hCG detection. MD simulations were used to investigate the structure of the immobilized peptide templates within the context of pre-polymerization mixtures. Our findings strongly indicate that the chosen peptides have conformational properties that mimic the dynamic behavior of the epitopes in the full protein, thereby supporting their potential to imprint molecular cavities suitable for hCG recognition. Template-monomer interactions were found to be driven by TBAm and NIPAm, with a minor contribution of BIS and negligible interactions with AA and APM monomers. These findings offer an opportunity for optimizing the pre-polymerization mixture by varying the monomer types or proportions to maximize the interactions with the template.

The synthesized MIPs have homogeneous size distributions and exhibit high binding affinity, and specificity toward the SV and PQ epitopes and hCG as shown through SPR and QCM analysis. The selectivity towards hCG over other proteins and potential interferants, including LH, was confirmed. Overall, the study suggests the feasibility of using imprinted nanogels as a class of cost-effective, stable alternatives to natural antibodies for hCG detection. In view of the receptor´s robustness and the demonstrated SPR sensors compatibility with multiple regeneration cycles, we foresee applications of the MIPs in reusable pregnancy tests and other hCG-related disease diagnostics.

## Supplementary Information

Supplementary Information.

## Data Availability

All data generated or analysed during this study are included in this published article [and its supplementary information files].
