# Supplementary material for: Development of molecularly imprinted polymers for the detection of human chorionic gonadotropin
Source: Sci Rep. 2025 Mar 26;15:10436. doi: 10.1038/s41598-025-94289-3 (PMC11947253; doi:10.1038/s41598-025-94289-3)
Supplement: Supplementary file 1 — Supplementary Information. [file 41598_2025_94289_MOESM1_ESM.pdf]

Supporting Information

Development of Molecularly Imprinted Polymers for the Detection of Human Chorionic Gonadotropin

Radvile Zubryté, Liliia Mavliutova, Yadiris García, Mark V. Sullivan, Nicholas W. Turner, Francesco Patitucci, Laura C. Polania, Verónica A. Jiménez, Robert Porter, Alice Mattsson and Börje Sellergren*

Supporting Tables

Table S1. Epitope properties

Sequence	Sequence length	Hydrophobicity	GRAVY	Mw average (g/mol)	Theoretical pI
**SIRLPGCPRGVNPVV**	15	28	0.30	1564	10.9
**PGPSDTPILPQ**	11	23	-0.64	1121	3.1

a) https://www.thermofisher.com/se/en/home/life-science/protein-biology/peptides-proteins/custom-peptide-synthesis-services/peptide-analyzing-tool.html

Table S2. Functional monomer feed ratio (mole %) used for MIP synthesis.

Entry	NIPAm	BIS	TBAm	APM	AAc	FITC-AAm
**MIP-SV**	45	5	40	5	5	0.8 mg
**MIP-PQ**	48	2	40	10	0	0.4 mg

Supporting Figures

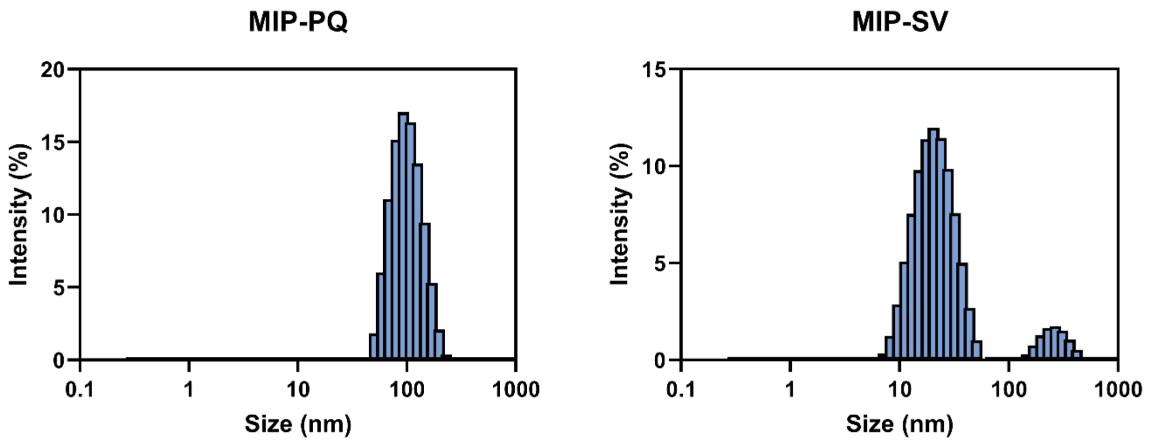

Figure S1. DLS size distribution of MIP-SV and MIP-PQ.

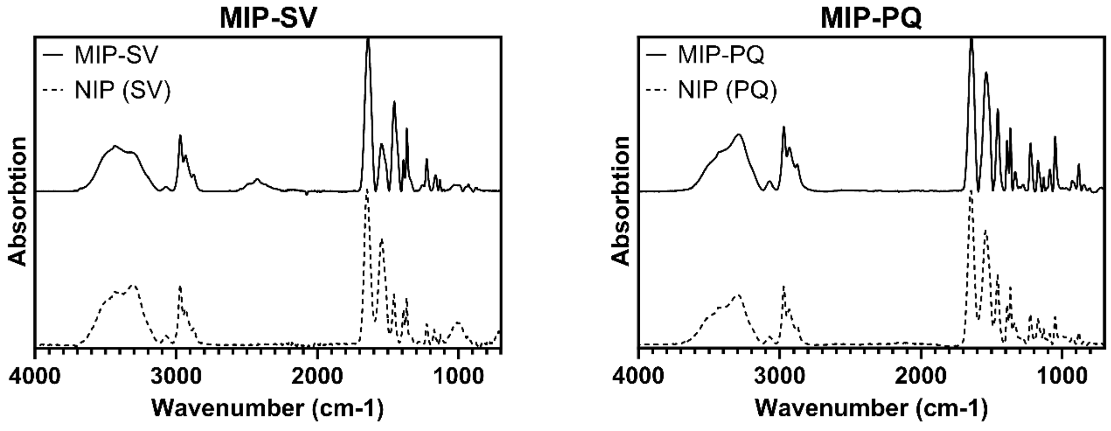

Figure S2. Fourier transform infrared (FTIR) spectroscopy of MIP-SV and MIP-PQ and corresponding NIPs.

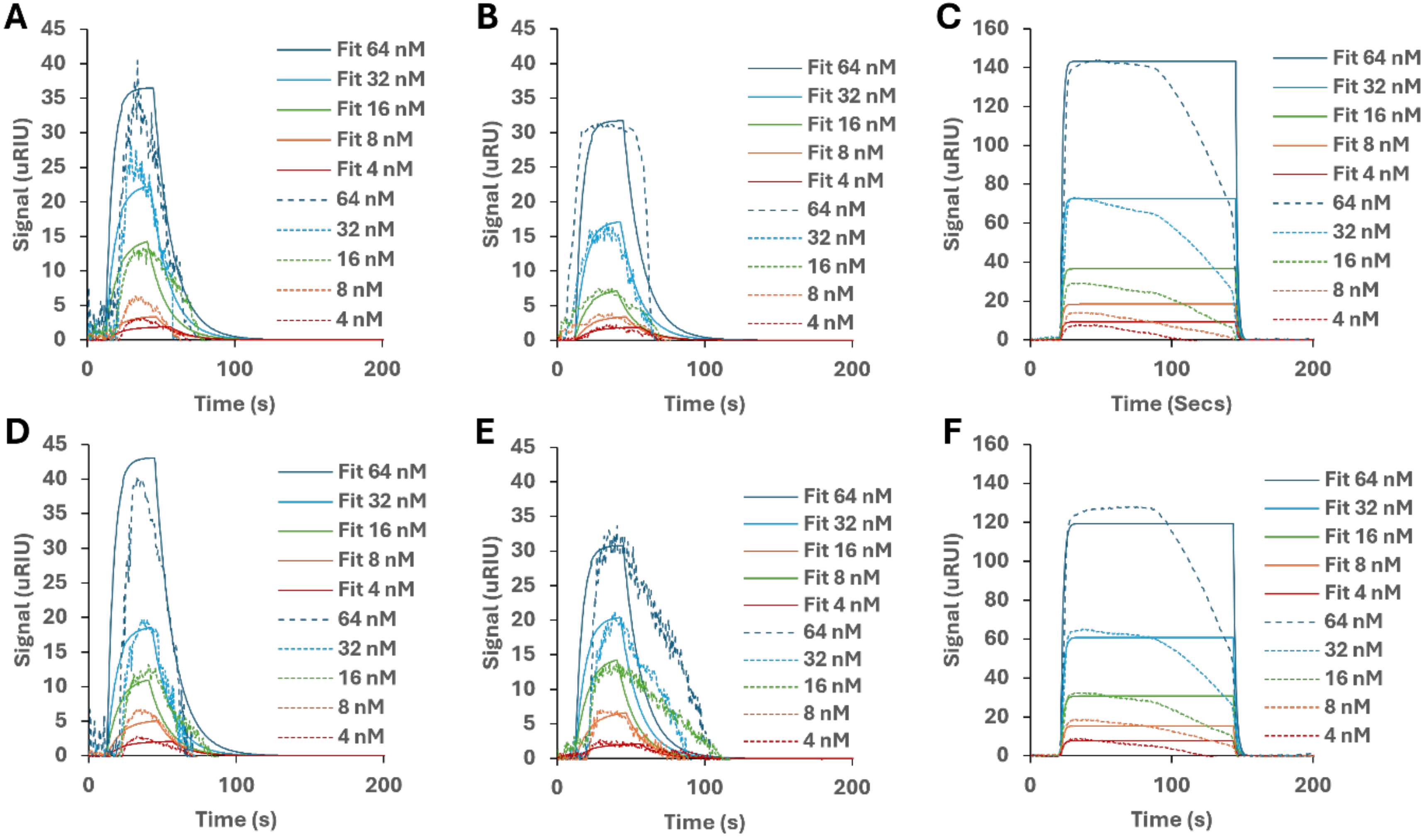

Figure S3. Representative fitted SPR curves showing the rebinding of the target and non-target peptides to the immobilized nanogel with five concentrations of the analyte in PBST. (A-C) SV, PQ and hCG binding to MIP-SV; (D-F) PQ, SV and hCG binding to MIP-PQ.

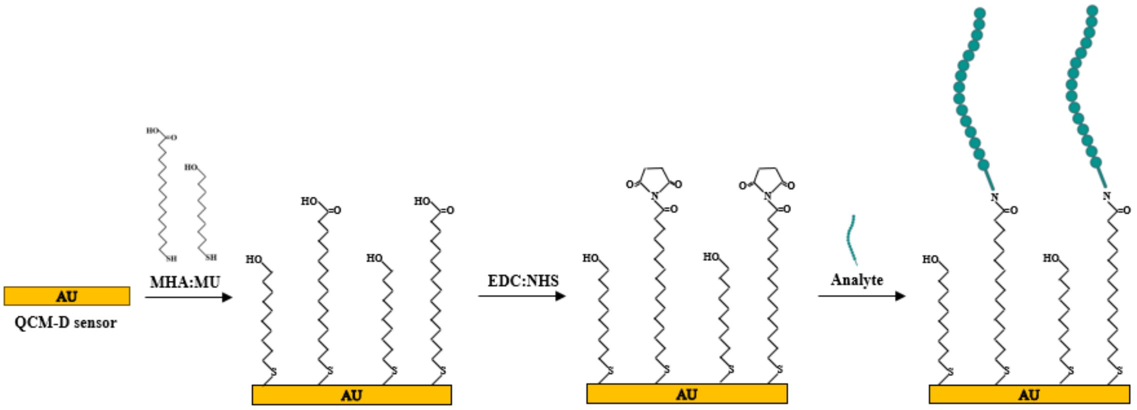

Figure S4. The process of QCM sensor chip modification with the target peptide.

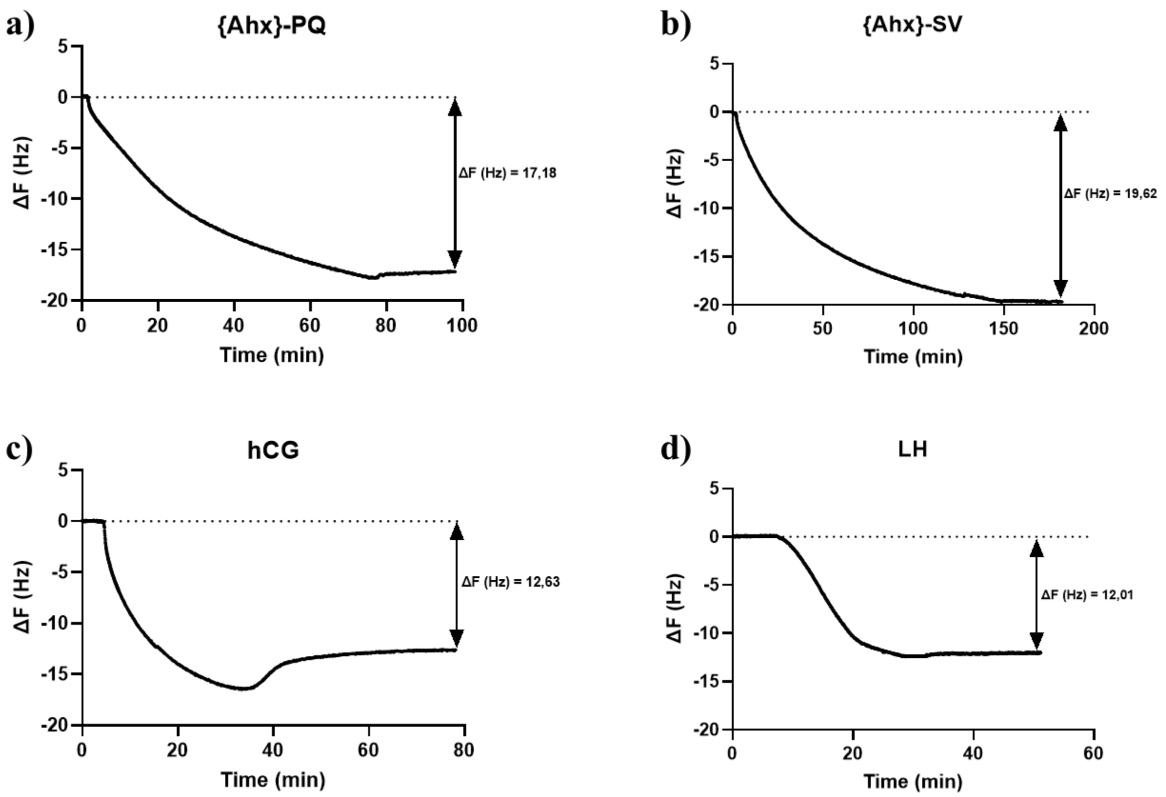

Figure S5. Analyses of QCM-D sensor modification with a) Ahx-PQ, b) Ahx-SV, c) hCG and d) LH. Ahx-PQ and Ahx-SV are the PQ and SV peptides conjugated with the N-acetylated-6-aminohexanoyl (Ahx) moiety at the N-termini.
